# Aphthous Fever in a Fœtus

**Published:** 1896-08

**Authors:** 


Aphthous Fever in a Fœtus. Districts-Veterinarian Bodenmueller discovered that the virus of aphthous fever can be transferred by the congenital blood to the embryo. An outbreak of aphthous fever occurred in a stable containing twelve cows, of which one was about to calve in a few days. On the fifth day the cow gave birth to a normal calf, but showing in mouth and between the toes the characteristic symptoms of aphthous fever. After the lapse of a few hours the calf died.—*Wochenschrift fuer Thierheilkunde.*

